# *QuickStats:* Percentage* of Adults Aged ≥50 Years with Osteoporosis,^†^ by Race and Hispanic Origin^§^ — United States, 2017–2018

**DOI:** 10.15585/mmwr.mm7019a5

**Published:** 2021-05-14

**Figure:**
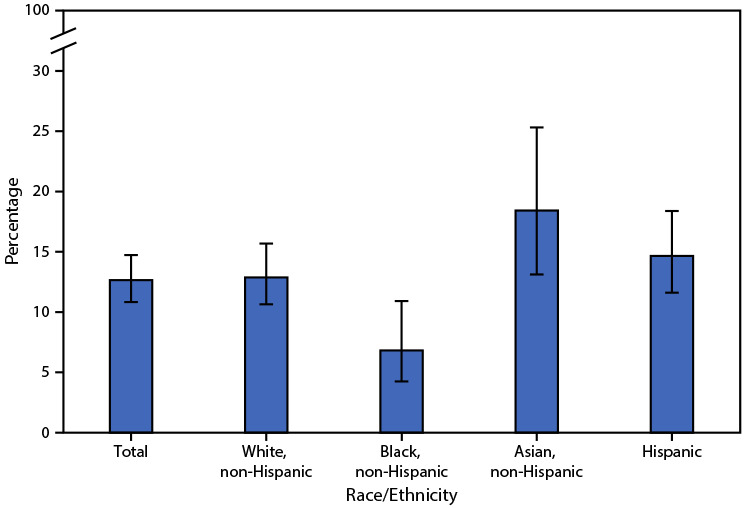
During 2017–2018, the age-adjusted prevalence of osteoporosis among adults aged ≥50 years was 12.6%. A lower percentage of non-Hispanic Black adults (6.8%) had osteoporosis compared with non-Hispanic White adults (12.9%), non-Hispanic Asian adults (18.4%), and Hispanic adults (14.7%). The observed differences among non-Hispanic White, non-Hispanic Asian, and Hispanic adults did not reach statistical significance.

**Sources:** Sarafrazi N, Wambogo EA, Shepherd JA. Osteoporosis or low bone mass in older adults: United States, 2017–2018. National Center for Health Statistics (NCHS) data brief, no. 405. https://www.cdc.gov/nchs/products/databriefs/db405.htm; NCHS, National Health and Nutrition Examination Survey (NHNES) data, NHNES 2017–2018. https://www.cdc.gov/nchs/nhanes.htm

